# Household Food Insecurity and Mental Health Among Teenage Girls Living in Urban Slums in Varanasi, India: A Cross-Sectional Study

**DOI:** 10.3390/ijerph15081585

**Published:** 2018-07-26

**Authors:** Divya Rani, Jitendra Kumar Singh, Dilaram Acharya, Rajan Paudel, Kwan Lee, Shri Prakash Singh

**Affiliations:** 1 Department of Community Medicine, Institute of Medical Sciences, Banaras Hindu University, Varanasi 221005, India; divyarani.bhu@gmail.com (D.R.); drspsingh_vns@yahoo.com (S.P.S.); 2 Department of Community Medicine and Public Health, Janaki Medical College, Tribhuvan University, Janakpur 44618, Nepal; jsingdj@gmail.com; 3 Department of Preventive Medicine, College of Medicine, Dongguk University, Gyeongju 04620, Korea; dilaramacharya123@gmail.com; 4 Department of Community Medicine and Public Health, Institute of Medicine, Tribhuvan University, Kathmandu 44600, Nepal; paudel.rajan@gmail.com; 5 Department of Community Medicine, Kathmandu University, Devdaha. Medical College and Research Institute, Rupandehi 32907, Nepal; 6 Health Foundation Nepal (HFN), Kathmandu 44600, Nepal

**Keywords:** cross-sectional study, household food insecurity, mental health, teenage girls, urban slums, India

## Abstract

This study was undertaken to investigate the relation between household food insecurity and mental health problems in teenage girls living in urban slums. This community-based cross-sectional study was conducted in 5 urban slums in Varanasi, India, between September 2016 and July 2017. A probability proportion to size (PPS) method was employed to select 5 of 210 urban slums at a first stage, and in the second stage, 418 teenage girls were chosen randomly from selected households. The Household Food Insecurity Access Scale (HFIAS) and mental health inventory tools were employed to assess food insecurity and mental health status. Multivariable logistic regression analysis with at a 95% confidence interval (CI) was used to assess the association between household food insecurity and mental health status. Of 418 respondents, 47.6% were food insecure; 64.1%, 57.7%, and 58.4% had high levels of anxiety, depression, or psychological distress, respectively; and 57.2% exhibited a medium level of loss of behavioral control. Furthermore, teenage girls from food insecure households were more likely to have high levels of anxiety, depression, loss of behavioral control and psychological distress than those living in food secure households. This study shows food insecurity is independently associated with mental health problems among teenage girls. Food insecurity in Indian slums should be addressed by specific public health intervention programs that provide access to sufficient safe, nutritious food.

## 1. Introduction

Food insecurity (FI) is a condition in which the availability of nutritionally adequate and safe foods or the ability to acquire such foods in socially acceptable ways is limited or uncertain [1,2]. Despite substantial improvements in food production over recent decades, about 795 million people worldwide remain food insecure [3], and FI is of growing concern because poor nutritional status affects the physical and mental health of children and adolescents in the developing and developed world [4,5,6,7,8,9]. Reports on food insufficient households frequently report reduced intake of adequate and nutritious food items [10,11,12,13], poor health, poor functional health, restricted activity, chronic medical conditions, and high rates of major depression and distress [14]. Household food insecurity is a mounting public health problem in low-income households [15] and is known to be significantly positively associated with lower income and education [12], higher family size and food prices [16], and lower quality of life [17].

Poor mental health is a serious public health issue, especially among children and adolescents. Mental health refers to a state of well-being in which the individual realizes his or her own abilities, can cope with the normal stresses of life, can work productively and fruitfully, and is able to make a contribution to his or her community [18]. The World Health Organization (WHO) reported that in 2014 approximately 20% of the world’s children and adolescents suffered from mental disorders or problems [19]. Adolescence is critical in terms of the initiation of mental illness, especially the late teenage period [20,21]. Mental health and well-being among young people has been found to be positively associated with self-esteem, attendance at school, educational achievement as well as future health and opportunities [22]. Furthermore, suicide is the major cause of death among adolescents and is often the result of mental disorders [23]. Additionally, mental disorders contribute substantially to health care costs and productivity [24]. 

A number of well-designed studies on the association between food insecurity and mental illness among children and adolescents [7,9,25,26], pregnant women [27], adults with disability [28] have concluded that food insecurity is a potential predictor of poor mental health status. In addition, food insecure females were found to have higher odds of having mental disorders compared with male counterparts [29]. However, there is a literature gap in the effort to demonstrate this relation in teenage girls living in slum areas, although about a billion people, or 32% of the world’s urban population are estimated to live in slums [30], and this has been projected to increase to 2 billion during the next 30 years [30,31]. 

In the current study setting of the Varanasi district of India, about 38% of the inhabitants are urban poor and slum dwellers, and 227 slums are distributed throughout the district. People living in the urban slums of Varanasi district have a low quality of life; 34% live below the poverty line, 35% have no access to government health care facilities, and 17% depend on traditional healers for health care services [32,33]. Moreover, food insecurity has been a huge public health problem in India, especially among the urban poor and those living in slum areas [34,35]. With these backgrounds, the present study aimed to determine the nature of the association between household food insecurity and mental health status among teenage girls living in slum areas of the Varanasi district.

## 2. Materials and Methods

### 2.1. Ethics

The study protocol was approved by the Ethical Committee of Banaras Hindu University, India (approval number: ECR/526.Inst/UP/2014 Dt.31.1.14). The study purpose and the procedure used for collecting information were clearly explained to each participant and they were all informed that they were free to leave the study at any time without prejudice. Written informed consent was obtained from all study participants. Privacy and confidentiality were maintained throughout and personal identifiers were removed prior to the data analysis.

### 2.2. Study Design and Setting 

The present study was conducted using a community-based, cross-sectional design between September 2016 and July 2017 in 5 of 210 urban slums in Varanasi, India. Varanasi (also known as Kashi or Banaras) is one of the oldest cities in the world with a continuous history spanning 3000 to 5000 years, and is also regarded as the cultural and spiritual capital of India. This urban area is located at 25°14’ N/82°56’ E/83°03’ E in a crescent-shaped region of the Ganga river, in which slums have grown continuously since 1941. Inhabitants of these slums lack amenities such as adequate housing, electricity and safe drinking water. About 38% of the inhabitants of Varanasi are urban poor and slum dwellers, and 227 slums are distributed throughout the city [32,33,36].

### 2.3. Sampling 

We used a stratified two-stage probability sampling design to select community-based samples. Initially, 5 slums (enumeration areas) were selected from 210 based on considerations of slum category stratified by population with probability proportion to size (PPS). During the second sampling stage, households were systematically selected from a list obtained from the Varanasi Slum Profile at a Glance report 2011 [36]. Girls (aged 13 to 19 years) were randomly chosen from selected households. Only one adolescent girl (the oldest when there was more than one) was selected from each household by using a random number table. Sample size was estimated using the following formula: N = (Z1 − α/2)2P × Q/L2, assuming a mental health issue prevalence of 50% and a precision of 10%. As a result, the required sample size was estimated to be 418. To test the adequacy of the sample size, we evaluated the statistical power of an existing study through post-hoc power calculations. Power calculations were made by comparing proportions of different outcome variables (anxiety, depression, loss of behavioral control, and psychological distress) between food secure and insecure groups. These showed an α of 0.05, and all comparison was adequately powered with the numbers that were recruited for the study.

### 2.4. Selection of Study Participants 

Of the 440 teenage girls invited to participate in the study, 418 agreed to participate (a response rate of 94.56%). Local community health volunteers in areas, such as *Aaganwadi/Trained dais*, assisted with the recruitment process. Adolescent girls with mental illness, such as intellectual disability, developmental delay, autism, or any other condition that inhibited communication or the ability to participate in the study were not included.

### 2.5. Data Collection

We employed five medical personnel who had graduated in psychiatric nursing as research assistants. Prior to data collection, they were given two days’ training about research methods, survey instruments, obtaining informed consent, and data collection. The principle investigator and two of the co-investigators monitored research assistants to ensure that households and study participants were appropriately selected in accordance with the sampling design to increase the quality of the study. All surveys and consent forms were translated into the local language (Hindi) and then back-translated into English to ensure translations were accurate. The survey questionnaires consisted of three parts: (1) personal profile (socio-demographic and socio-economic characteristics), (2) household food security, and (3) mental health state-related questionnaires.

### 2.6. Food Insecurity

The Household Food Insecurity Access Scale (HFIAS), which was developed by the Food and Nutrition Technical Assistance Project, Academy for Educational Development, USA, in August 2007, was used to assess food security. This scale classifies individuals into four levels of household food insecurity, that is, food-secure or mildly, moderately or severely food-insecure over the previous 30 days based on subject recall period. Respondents were asked to respond to these questions (with yes or no response options) based on their experiences during the previous four weeks (30 days). When a respondent answered ‘yes’ to a question, a frequency-of-occurrence question was asked, and responses were as follows: rare (once or twice; response code 1), sometimes (three to 10 times; response code 2), or often (more than 10 times; response code 3).

HFIAS scores were used as continuous measures of degrees of household food insecurity and were calculated by summing scores for frequency-of-occurrence questions for each household. The maximum score for a household was 27, meaning the individual responded to all nine frequency-of-occurrence questions with a response code of 3 and the minimum score was 0, which meant the individual answered “no” to all frequency-of-occurrence questions. Thus, higher scores indicated greater food insecurity [37]. In addition to the nine food insecurity occurrence questions, nine frequency of occurrence questions were asked to determine the frequency of the condition. These questions were asked to determine how frequently the condition mentioned in the occurrence question occurred. The questionnaire addressed major components of food insecurity, such as, (1) anxiety and uncertainty about household food supply, (2) insufficient quality of food (included food varieties and preferences), and (3) insufficient food intake and its physical consequences.

### 2.7. Mental Health Assessment 

The Mental Health Inventory was used to assess the mental health status of the respondents. This questionnaire contained 38 items, which were measured in terms of frequency or intensity. Each item included a description of a particular symptom or state of mind and respondents were asked to indicate degrees on a scale based on experiences over the previous months. This tool was adopted to recognize cognitive, general positive effects, emotional ties and levels of anxiety, depression, and emotional/behavioral control among children aged ≥ 13 years. In the present study, we used four components of the Mental Health Inventory, namely, anxiety, depression, loss of behavioral control, and psychological distress. Each component was further categorized as low, medium or high. The scores were classified as follows: (1) for anxiety, low 9–24, medium 25–39, and high 40–54; (2) for depression, low 4–10, medium 11–16, and high 17–23; (3) for loss of behavior control, low 9–22, medium 23–38, and high 39–53; and (4) for psychological distress, low 24–60, medium 61–100, and high 105–142 [38,39,40]. In our study findings for all components of mental health (anxiety, depression, loss of behavior control, and psychological distress), the proportion of participants in the low category was either very small or zero. Therefore, we merged low categories into the medium category, and considered one category “low/medium”, and logistic regressions were run (low/medium vs high) as binary outcomes.

### 2.8. Statistical Analysis

Data were entered into Epi Data 3.1 and then transferred to SPSS for Windows Ver. 22.0 (SPSS Inc. Chicago, IL, USA). Multivariate logistic regression was used to assess the association between food insecurity and mental health status. Key components of mental health outcomes included: (1) anxiety, (2) depression (3) loss of behavioral control, and (4) psychological distress. Socio-demographic characteristics were adjusted in all multivariable logistic regression models to estimate the influence of food insecurity on mental health. Covariates included in the multivariate analyses were: (1) age (13–16 years, 17–19 years); (2) religion (Hindu, others); (3) caste: SC (schedule caste: untouchable lower castes)/ST (schedule tribes: origin of residence in forest especially not being involved in agricultural work), OBC (other backward caste), general (upper caste group); (4) type of family (nuclear, joint); (5) head of family (male, female); (6) no. of people in family (>4, ≤4); (7) no. of siblings (>2, ≤2); (8) years resident in a slum (>30 or ≤30 years); (9) education level (primary and lower, secondary and above); (10) education of mother (primary and lower, secondary and above); (11) education of father (primary and lower, secondary and above); (12) occupation (working, student); (13) occupation of mother (working, home maker); (14) occupation of father (service/business, agriculture/labor); (15) family income: first tercile (≤5000 Indian rupees/month), second tercile (5001–10,666 Indian rupees/month), and third tercile (≥10,667 Indian rupees/month), as previously described [41]. Regression diagnostic procedures yielded no evidence of multicollinearity or overly influential outliers in any model. Results are expressed as odds ratios (ORs) with 95% confidence intervals for binary outcomes of mental health. All tests were two tailed and *p* values of <0.05 were deemed significant.

## 3. Results

### 3.1. Household Food Security and Mental Health Status Levels Among Study Participants

For the 418 girls, nearly half of households (47.6%) were food insecure; nearly two fifths of households (37.6%) were moderately insecure; and 4.5% were severely food insecure. Table 1 shows that most of the study subjects had a high level of anxiety (64.1%), depression (57.7%), psychological distress (58.4%), and 57.2% had a medium level of loss of behavioral control.

### 3.2. Socio-Demographic Characteristics and Mental Health Status 

Of the 418 study subjects, slightly more than half (53.9%) were 17–19 years old, the majority (77.6%) were of OBC and SC/ST ethnicity, lived in a nuclear family (83.4%), had >4 family members (86.9%), and had >2 siblings (80.9%). Almost all (98.5%) households had resided in a slum for >30 years. Slightly more than half (53.3%) of the study subjects had secondary and higher level of education, while the majority of mothers (87.1%) and fathers (60.6%) had an education level of primary and less. More than three fourths (78.9%) of the study subjects were students. Regarding their parents’ occupations, 73.2% of mothers were home makers and 50.9% of fathers worked in agriculture or as laborers. The majority of family incomes were in the first tercile (Table 2). Levels of a number of characteristics of poor mental health status, that is, anxiety, depression, loss of behavioral control, and psychological distress, were significantly higher among girls from food insecure households (Table 3). 

### 3.3. Associations between Mental Health Status and Food Insecurity and Socio-Economic Characteristics

Table 4 presents the results of unadjusted logistic regression analyses of relations between mental health status and household food insecurity and subject characteristics. Food insecurity, study subjects aged 13–16, >4 family members, >2 siblings, a mother and father educated to primary level or lower, a working mother, a father working in labor/agriculture, working herself, and family income in the first and second terciles were significantly associated with high anxiety. In addition, food insecurity, SC/ST caste, >2 siblings, a father and mother educated to primary level or lower, a working mother, a father working in agriculture or as a laborer, and a family income in the first and second terciles were significantly associated with high depression. 

Similarly, food insecurity, a father and mother educated to primary level or lower, working herself, a father working in agriculture or as a laborer, and a family income in the first and second terciles were significantly associated with high loss of behavioral control. In addition, food insecurity, >2 siblings, participant and her mother working outside, a father educated to primary level or lower, a father working in agriculture or as a laborer, and a family income in the first or second terciles were significantly associated with high psychological distress.

Results obtained using the final multivariate logistic regression analysis model for relations between mental health status and household food insecurity and subject characteristics are shown in Table 5.

Teenage girls of SC/ST ethnicity had higher odds of having high levels of anxiety (adjusted odds ratio (AOR) = 3.15, 95% CI = (1.10–9.01)), depression (AOR = 2.81, 95% CI = (1.00–7.98)), and loss of behavioral control (AOR = 2.21, 95% CI = (1.00–5.04)). Subjects with a father educated to the primary level or lower had higher odds of having high depression (AOR = 2.07, 95% CI = (1.08–3.95)) and suffer from high loss of behavioral control (AOR = 2.20, 95% CI = (1.21–3.99)). Subjects working outside were found to have higher odds of having high levels of anxiety (AOR = 3.24, 95% CI = (1.25–8.37)), high depression (AOR = 2.93, 95% CI = (1.08–7.90)), high loss of behavioral control (AOR = 6.85, 95% CI = (2.21–21.17)) and of high psychological distress (AOR = 3.15, 95% CI = (1.25–7.95)). Subjects with a working mother had a higher odds of having high anxiety level (AOR = 2.04, 95% CI = (1.00–4.14)). Subjects from a family with an income in the first tercile had higher odds of having to exhibit high anxiety (AOR = 2.65, 95% CI = (1.26–5.59)) and high depression (AOR = 2.81, 95% CI = (1.34–5.89)), and subjects from a family with an income in the second tercile were found to have higher odds of having high depression (AOR = 2.70, 95% CI = (1.00–7.76)) and high psychological distress (AOR = 2.66, 95% CI (1.00–7.23)) than their counterparts with a family income in the third tercile. In addition, girls aged 13 to 16 years were found to have lower odds of having high levels of anxiety (AOR = 0.31, 95% CI = (6.08–39.13)), high depression (AOR = 0.40, 95% CI = (0.19–0.83)), and high psychological distress (AOR = 0.51, 95% CI = (0.28–0.99)) than girls aged 17 to 19 years.

Finally, we examined the association between household food insecurity and mental health status after controlling for socio-economic characteristics (Table 5). Girls from food insecure households were found to have higher odds of having high levels of anxiety (AOR = 15.42, 95% CI = (6.08–39.13)), high depression (AOR = 13.69, 95% CI = (5.89–31.79)), high loss of behavioral control (AOR = 6.84, 95% CI = (3.24–14.43)) and high psychological distress (AOR = 8.70, 95% CI = 4.01–18.87)) than those living in food secure households.

## 4. Discussion

In this community-based cross-sectional study, we investigated the association between household food insecurity and mental health status in teenage girls living in Indian urban slums. We found that nearly a half of households suffered food insecurity, and that nearly two fifths and 4.5% of households suffered moderately and severely, respectively, from food insecurity, which are higher levels than have been reported in studies conducted in the USA [42], Canada [25], France [7], Ethiopia [27], or South Africa [43]. We attribute this greater level of food insecurity to the slum setting of the present study, as other studies were conducted in non-slum areas. Other possible reasons include family sizes and socio-economic variations. However, proportions of household food insecurity found in the present study are similar to those reported in an Indian study conducted in an urban slum in northern India [34]. 

We found that the majority of our study subjects had high levels of anxiety, depression, and psychological distress, and that slightly more than half had a medium-level of loss of behavioral control. In previous studies, living in urban slum households with a poor quality of life was suggested to influence the mental health status of teenage girls [32,33]. Many studies supported that those living in poverty or lower socioeconomic status, disabled, housewives living in slums [44,45], and those living in the most deprived areas [46] are at risk of poor mental health status.

Teenage girls working outside the home were estimated to have higher odds of high levels of anxiety, depression, behavioral control loss, and psychological distress. Although employment may have a protective effect on mental health, our study participants were from urban slums and performed menial daily work [47]. Furthermore, adolescents must work as adults and, thus, leave the education system [48,49,50], and as a result, mental health problems are common among working adolescents [48,51,52].

The present study also shows teenage girls from socio-economically disadvantaged groups (schedule caste/schedule tribe) with a low family income (first and second terciles) were more likely to have mental health issues, which accords with previous studies [8,53,54]. For example, a systematic review paper reported socioeconomically disadvantaged adolescents with a persistent low socioeconomic status were more likely to develop mental health problems [53], and Dashiff et al. [54] reported poverty has direct effects on adolescent mental health. Similarly, we found a paternal education of primary or lower places teenage daughters at risk of mental health problems. Avci et al. reported fathers with a primary school level of education had significantly higher emotional and behavioral problem scores than fathers educated to higher levels [55]. 

Interestingly, our study revealed girls in their early teens (13–16 years) tended to have lower levels of anxiety, depression, and psychological distress than older teenage girls (17–19 years). Although most mental disorders begin between the ages of 12–24 years, they are often detected in later life [56]. A study conducted in the USA reported that around a half of life-long mental disorders start before 14 years of age [57], and the Australian National Survey of Mental Health and Wellbeing (NSMHWB) found that 27% of 18- to 24-year-olds had a mental disorder [58]. Based on these reports, emphasis should be given to the importance of early intervention to improve mental health outcomes in adolescents and young adults.

After controlling for potential confounders, multivariate analysis showed household food insecurity was strongly associated with high anxiety, high depression, high loss of behavioral control and high psychological distress, which are consistent with the findings of many other studies [7,8,9,26,43]. Although no casual mechanism between FI and negative mental health has been established for adolescents, it has been suggested that uncertainty regarding the maintenance of food supplies generates stresses that might contribute to negative outcomes [59,60]. Additionally, FI may augment socioeconomic discrepancies within households and communities and trigger or exacerbate cultural sensitivities which, in turn, might adversely influence mental health [61].

This study has some specific strengths. In particular, it is the first study to examine associations between food insecurity and the mental health statuses of teenage girls living in the urban slums of northern India. In addition, methodologically sound tools were used to assess household food insecurity [37] and mental health statuses [38]. However, the study also has a number of limitations. First, we did not compare degrees of associations between teenage girls from households with marginal, low or very low food security and mental health, or between teenage girls and household heads; and because this study was conducted on a homogenous population of teenage girls living in slum areas we were could not able to compare relations between teenage girls living in slum areas and non-slum areas or compare between male and female teenagers. Second, we conducted the study in a small sample size that might suffer from poor external validity. Third, in the statistical analyses, we treated low and medium food insecurity as one category. Fourth, because of the cross-sectional nature of the study, we report only associations as we could not determine causalities. Fourth, our results do not rule out the possibility that poor mental health status among adolescent girls causes household food insecurity [62]. Finally, some potential confounders, such as, exposure to violence, not adjusted for in the present study, may have impacted mental health outcomes [63,64,65].

## 5. Conclusions

Approximately half of our teenage girls were found to be food insecure and to have high levels of anxiety, depression, and psychological distress, and medium loss of behavioral control, which are much greater effects than those reported in the developed world. In our study group, food insecurity was found to be independently associated with mental health status. Our findings show this higher burden of common mental health problems among teenage girls in Indian slums requires specific public health interventions that include access to sufficient, safe and nutritious food and socio-economic improvements to be instituted. 

## Figures and Tables

**Table 1:** Mental health status (anxiety, depression, loss of behavioral control, and psychological distress) of the study subjects.

Mental Health Status	Level	Score	No. of Cases	Percentage
Anxiety	Low	9–24	6	1.4
	Medium	25–39	144	34.4
	High	40–54	268	64.1
Depression	Low	4–10	13	3.1
	Medium	11–16	164	39.2
	High	17–23	241	57.7
Loss of behavioral control	Low	9–22	1	.2
	Medium	23–38	239	57.2
	High	39–53	178	42.6
Psychological distress	Low	24–60	1	0.2
	Medium	61–100	173	41.4
	High	105–142	244	58.4

**Table 2:** Associations between household food insecurity and socio-demographic characteristics.

Socio-Demographic Characteristics	Category	Food Insecure *n* = 199, (%)	Food Secure *n* = 219, (%)	Test of Significance	*p*-Value
Age				χ2 = 5.68, df = 1	*p* = 0.017
	13–16 years	115 (57.8)	101 (46.1)		
	17–19 Years	84 (42.2)	118 (53.9)		
Religion				χ2 = 0.82, df = 1	*p* = 0.365
	Hindu	194 (97.5)	210 (95.9)		
	Muslim	5 (2.5)	9 (4.1)		
Caste/Ethnicity				χ2 = 24.79, df = 2	*p* < 0.001
	General (upper caste group)	11 (5.5)	49 (22.4)		
	OBC (other backward caste)	83 (41.7)	74 (33.8)		
	SC (schedule caste)/ST (schedule tribes)	105 (52.8)	96 (43.8)		
Type of family				χ2 = 5.02, df = 1	*p* = 0.025
	Nuclear	166 (83.4)	163 (74.4)		
	Joint	33 (16.6)	56 (25.6)		
Head of family				χ2 = 1.00, df = 1	*p* = 0.987
	Female	21 (10.6)	23 (10.5)		
	Male	178 (89.4)	196 (89.5)		
Number of people in family				χ2 = 11.69, df = 1	*p* < 0.001
	≤4	26 (13.1)	58 (26.5)		
	>4	173 (86.9)	161 (73.5)		
Number of siblings				χ2 = 11.47, df = 1	*p* < 0.001
	≤2	38 (19.1)	74 (33.8)		
	>2	161 (80.9)	145 (66.2)		
Year resident in a Slum				χ2 = 3.23, df = 1	*p* = 0.072
	≤30 year	196 (98.5)	209 (95.4)		
	>30 year	3 (1.5)	10 (4.6)		
Education of subject				χ2 = 20.35, df = 1	*p* < 0.0001
	Primary and lower	93 (46.7)	56 (25.6)		
	Secondary and more	106 (53.3)	163 (74.4)		
Education of mother				χ2 = 61.34, df = 1	*p* < 0.0001
	Primary and lower	169 (87.1)	109 (50.9)		
	Secondary and more	25 (12.9)	105 (49.1)		
Education of father				χ2 = 38.38, df = 1	*p* < 0.00001
	Primary and lower	106 (60.6)	59 (28.9)		
	Secondary and more	69 (39.4)	145 (71.1)		
Occupation of subjects				χ2 = 16.61, df = 1	*p* < 0.001
	Student	157 (78.9)	203 (92.7)		
	Working outside (service, business, labor)	42 (21.1)	16 (7.3)		
Occupation of mother (*n* = 408)				χ2 = 4.84, df = 1	*p* = 0.028
	Home maker	142 (73.2)	176 (82.2)		
	Working outside (service, business, labor)	52 (26.8)	38 (17.8)		
Occupation of father (*n* = 379)				χ2 = 44.59, df = 1	*p* < 0.001
	Service/business	86 (49.1)	169 (82.8)		
	agriculture/labour	89 (50.9)	35 (17.2)		
Family income				χ2 = 220.79, df = 2	*p* < 0.00001
	1st tercile	141 (70.9)	15 (6.8)		
	2nd tercile	52 (26.1)	71 (32.4)		
	3rd tercile	6 (3.0)	133 (60.7)		

**Table 3:** Associations between household food insecurity and mental health status.

Mental Health Status	Level	Food Insecure *n* = 199, (%)	Food Secure *n* = 219, (%)	Test of Significance *	*p*-Value
Anxiety				χ^2^ = 103.8, df = 1	*p* < 0.0001
	Low	1 (0.5)	5 (2.3)		
	Medium	20 (10.1)	124 (56.6)		
	High	178 (89.4)	90 (41.1)		
Depression				χ^2^ = 126.5, df = 1	*p* < 0.0001
	Low	2 (1.0)	11 (5.0)		
	Medium	25 (12.6)	139 (63.5)		
	High	172 (86.4)	69 (31.5)		
Loss of behavioral control				χ^2^ = 97.1, df = 1	*p* < 0.0001
	Low	1 (0.5)	0 (0.0)		
	medium	63 (31.7)	176 (80.4)		
	High	135 (67.8)	43 (19.6)		
Psychological distress				χ^2^ = 108.1, df = 1	*p* < 0.0001
	Low	1 (0.5)	0 (0.0)		
	medium	29 (14.6)	144 (65.8)		
	High	169 (84.9)	75 (34.2)		

* Since figures in the Low category were small, it was merged with the medium category and chi square values were obtained by these categories as one category at 1 degree of freedom of one.

**Table 4:** Logistic regression analyses of relations between mental health status and household food insecurity and subject characteristics (unadjusted).

Characteristics	High Anxiety OR (95% CI)	High Depression OR (95% CI)	High Loss of Behavioral Control OR (95% CI)	High Psychological Distress OR (95% CI)
**Food insecurity**				
No	1.00 (Ref.)	1.00 (Ref.)	1.00 (Ref.)	1.00 (Ref.)
Yes	12.27 (7.44–20.23)	13.89 (8.60–22.42)	8.35 (5.29–13.16)	10.86 (6.83–17.28)
**Age**				
17–19 years	1.00 (Ref.)	1.00 (Ref.)	1.00 (Ref.)	1.00 (Ref.)
13–16 years	0.61 (0.41–0.92)	0.71 (0.48–1.05)	0.85 (0.57–1.26)	0.73 (0.49–1.08)
**Religion**				
Others	1.00 (Ref.)	1.00 (Ref.)	1.00 (Ref.)	1.00 (Ref.)
Hindu	1.0 (0.33–3.06)	1.33 (0.43–4.05)	0.74 (0.24–2.25)	1.29 (0.42–3.93)
**Caste** **/Ethnicity**				
General (upper caste group)	1.00 (Ref.)	1.00 (Ref.)	1.00 (Ref.)	1.00 (Ref.)
OBC (other backward caste)	1.48 (0.76–2.86)	1.64 (0.87–3.11)	1.29 (0.71–2.35)	1.56 (0.83–2.93)
SC (schedule caste)/ST (schedule tribes)	1.78 (0.94–3.37)	2.05 (1.10–3.80)	1.75 (0.98–3.14)	1.71 (0.93–3.15)
**Type of family**				
Joint	1.00 (Ref.)	1.00 (Ref.)	1.00 (Ref.)	1.00 (Ref.)
Nuclear	1.06 (0.64–1.73)	0.92 (0.57–1.48)	1.13 (0.70–1.81)	1.0 (0.62–1.61)
**Head of family**				
Female	1.00 (Ref.)	1.00 (Ref.)	1.00 (Ref.)	1.00 (Ref.)
Male	0.57 (0.30–1.08)	0.63 (0.34–1.19)	0.53 (0.26–1.04)	0.75 (0.40–1.41)
**Number of family member**				
≤4	1.00(Ref.)	1.00 (Ref.)	1.00 (Ref.)	1.00 (Ref.)
>4	1.75 (1.02–2.98)	1.41 (0.86–2.32)	1.45 (0.90–2.35)	1.55 (0.94–2.56)
**Number of siblings**				
≤2	1.00 (Ref.)	1.00 (Ref.)	1.00 (Ref.)	1.00 (Ref.)
>2	1.98(1.22–3.23)	1.80 (1.14–2.84)	1.50 (0.97–2.33)	1.83(1.15–2.89)
**Year of residence in a Slum**				
≤30 years	1.00 (Ref.)	1.00 (Ref.)	1.00 (Ref.)	1.00 (Ref.)
>30 years	3.16 (0.69–14.48)	1.67 (0.50–5.53)	1.59 (0.52–4.83)	1.62 (0.49–5.37)
**Education of subject**				
Secondary and above	1.00 (Ref.)	1.00 (Ref.)	1.00 (Ref.)	1.00 (Ref.)
Primary and lower	1.40 (0.92–2.12)	1.46 (0.97–2.18)	1.37 (0.91–2.07)	1.34 (0.89–2.01)
**Education of mother**				
Secondary and above	1.00 (Ref.)	1.00 (Ref.)	1.00 (Ref.)	1.00 (Ref.)
Primary and lower	2.95 (1.80–4.82)	3.19 (2.00–5.09)	2.35 (1.53–3.59)	2.73 (1.72–4.33)
**Education of father**				
Secondary and above	1.00 (Ref.)	1.00 (Ref.)	1.00 (Ref.)	1.00 (Ref.)
Primary and lower	2.87 (1.84–4.47)	3.47 (2.25–5.34)	3.36 (2.18–5.19)	2.93 (1.91–4.49)
**Occupation of subjects**				
Student	1.00 (Ref.)	1.00 (Ref.)	1.00 (Ref.)	1.00 (Ref.)
Working outside (service, business, labor)	4.60 (2.54–8.32)	4.37 (2.36–8.08)	7.92 (3.32–18.92)	4.11 (2.24–7.53)
**Occupation of mother**				
Home maker	1.00 (Ref.)	1.00 (Ref.)	1.00 (Ref.)	1.00 (Ref.)
Working outside (service, business, labor)	2.08 (1.29–3.35)	1.94 (1.21–3.12)	1.34 (0.83–2.17)	1.70 (1.06–2.72)
**Occupation of father**				
Service/business	1.00 (Ref.)	1.00 (Ref.)	1.00 (Ref.)	1.00 (Ref.)
Agriculture/labor	2.74 (1.74–4.30)	3.37 (2.15–5.27)	3.23 (2.01–5.18)	3.09 (1.98–4.83)
**Family income**				
3rd tercile	1.00 (Ref.)	1.00 (Ref.)	1.00 (Ref.)	1.00 (Ref.)
2nd tercile	2.36 (1.28–4.37)	2.61 (1.46–4.68)	1.93 (1.18–3.17)	2.45 (1.38–4.33)
1st tercile	9.26(5.22–16.42)	12.83 (7.28–22.63)	6.68 (3.97–11.24)	9.66 (5.58–16.72)

**Table 5:** Logistic regression analyses of household food insecurity and mental health status as determined by adjusted multivariate analysis.

Characteristics	High Anxiety	High Depression	High Loss of Behavioral Control	High Psychological Distress
	AOR (95% CI)	AOR (95% CI)	AOR (95% CI)	AOR (95% CI)
**Age**				
17–19 years	1.00 (Ref.)	1.00 (Ref.)	1.00 (Ref.)	1.00 (Ref.)
13–16 years	0.31 (0.15–0.64)	0.40 (0.19–0.83)	0.75 (0.41–1.37)	0.51 (0.28–0.99)
**Religion**				
Others	1.00 (Ref.)	1.00 (Ref.)	1.00 (Ref.)	1.00 (Ref.)
Hindu	2.32 (0.44–12.22)	1.63 (0.30–8.72)	2.99 (0.80–11.14)	1.25(0.25–6.08)
**Caste**				
General (upper caste group)	1.00 (Ref.)	1.00 (Ref.)	1.00 (Ref.)	1.00 (Ref.)
OBC (other backward caste)	2.27 (0.78–6.53)	1.75 (0.60–5.03)	1.42 (0.60–3.36)	2.01 (0.76–5.32)
SC (schedule caste)/ST (schedule tribes)	3.15 (1.10–9.01)	2.81 (1.00–7.98)	2.21 (1.00–5.04)	2.31 (0.89–5.97)
**Type of family**				
Joint	1.00 (Ref.)	1.00 (Ref.)	1.00 (Ref.)	1.00 (Ref.)
Nuclear	1.05 (0.47–2.33)	2.09 (0.93–4.68)	0.95 (0.47–1.93)	1.43 (0.67–3.02)
**Head of family**				
Female	1.00 (Ref.)	1.00 (Ref.)	1.00 (Ref.)	1.00 (Ref.)
Male	0.62 (0.15–2.46)	0.78 (0.19–3.08)	0.37 (0.10–1.28)	1.19 (0.31–4.53)
**Number of people in family**				
≤4	1.00 (Ref.)	1.00 (Ref.)	1.00 (Ref.)	1.00 (Ref.)
>4	1.41 (0.39–5.06)	2.59 (0.72–8.28)	1.41 (0.49–4.04)	1.55 (0.46–5.19)
**Number of siblings**				
≤2	1.00 (Ref.)	1.00 (Ref.)	1.00 (Ref.)	1.00 (Ref.)
>2	1.98 (0.67–5.85)	2.19 (0.73–6.55)	1.31 (0.52–3.31)	1.99 (0.70–5.63)
**Years of residence in a slum**				
≤30 year	1.00 (Ref.)	1.00 (Ref.)	1.00 (Ref.)	1.00 (Ref.)
>30 year	1.60 (0.19–13.18)	1.00 (0.16–6.15)	0.91 (0.22–3.72)	1.21 (0.22–6.69)
**Education of subject**				
Secondary and above	1.00 (Ref.)	1.00 (Ref.)	1.00 (Ref.)	1.00 (Ref.)
Primary and lower	1.37 (0.64–2.94)	1.20 (0.57–2.55)	1.59 (0.81–3.10)	1.52 (0.75–3.10)
**Education of mother**				
Secondary and above	1.00 (Ref.)	1.00 (Ref.)	1.00 (Ref.)	1.00 (Ref.)
Primary and lower	1.24 (0.53–2.88)	1.40 (0.60–3.24)	1.56 (0.77–3.15)	1.67 (0.75–3.67)
**Education of father**				
Secondary and above	1.00 (Ref.)	1.00 (Ref.)	1.00 (Ref.)	1.00 (Ref.)
Primary and lower	1.55 (0.80–2.98)	2.07 (1.08–3.95)	2.20 (1.21–3.99)	1.75 (0.94–3.26)
**Occupation of subjects**				
Student	1.00 (Ref.)	1.00 (Ref.)	1.00 (Ref.)	1.00 (Ref.)
Working outside (service, business, labor)	3.24 (1.25–8.37)	2.93 (1.08–7.90)	6.85 (2.21–21.17)	3.15 (1.25–7.95)
**Occupation of mother**				
Home maker	1.00 (Ref.)	1.00 (Ref.)	1.00 (Ref.)	1.00 (Ref.)
Working outside (service, business, labor)	2.04 (1.00–4.14)	1.44 (0.70–2.97)	1.03 (0.53–2.00)	1.42 (0.72–2.81)
**Occupation of father**				
Service/business	1.00 (Ref.)	1.00 (Ref.)	1.00 (Ref.)	1.00 (Ref.)
Agriculture/labour	1.22 (0.65–2.29)	1.35 (0.72–2.54)	1.69 (0.92–3.11)	1.36 (0.75–2.48)
**Family income**				
3rd tercile	1.00 (Ref.)	1.00 (Ref.)	1.00 (Ref.)	1.00 (Ref.)
2nd tercile	1.51 (0.49–4.62)	2.70 (1.00–7.76)	1.51 (0.60–3.76)	2.66 (1.00–7.23)
1st tercile	2.65 (1.26–5.59)	2.81 (1.34–5.89)	1.77 (0.83–3.74)	2.37 (1.17–4.81)
**Food insecurity**				
No	1.00 (Ref.)	1.00 (Ref.)	1.00 (Ref.)	1.00 (Ref.)
Yes	15.42 (6.08–39.13)	13.69 (5.89–31.79)	6.84(3.24–14.43)	8.70(4.01–18.87)
